# A Secure Transmission Scheme Based on Artificial Fading for Wireless CrowdSensing Networks

**DOI:** 10.3390/s18103500

**Published:** 2018-10-17

**Authors:** Zhi-Jiang Xu, Fang-Ni Chen, Yuan Wu, Yi Gong

**Affiliations:** 1 Zhijiang College, Zhejiang University of Technology, Shaoxing 312030, China; zyfxzj@zjut.edu.cn; 2 College of Information Engineering, Zhejiang University of Technology, Hangzhou 310023, China; iewuy@zjut.edu.cn; 3 Department of Electrical and Electronic Engineering, Southern University of Science and Technology, Shenzhen 518055, China; cfnini@163.com; 4 School of Information and Electronic Engineering, Zhejiang University of Science and Technology, Hangzhou 310023, China

**Keywords:** chaotic sequence, artificial fading, physical layer security, wireless crowdsensing networks

## Abstract

For secure transmission of low cost single antenna communication nodes in wireless crowdsensing networks under static channel, a physical layer communication scheme is proposed, where each digital modulated symbol is encrypted by a random key at the transmitter and decrypted with the same key at the receiver. The legal users exploit the synchronized chaotic sequence and the two-stage block interleaver to generate a complex random variable (random key), whereby its envelope obeys the Rayleigh distribution and its phase obeys the uniformly distribution. The modulated symbol is multiplied by the complex random variable (encryption) to imitate the Rayleigh fading of the channel at the transmitting end. The received symbol is divided by the identical complex random variable (decryption) to recover the transmitted message before the digital demodulation at the receiving end. Simulation results show that the bit error ratio (BER) performance of the legitimate users is consistent with the theoretical value of the Rayleigh fading channel, while the corresponding BER of the eavesdropper is too high (about 0.5) to intercept any information.

## 1. Introduction

With the rapid development during the past decades, wireless communication networks have been applied in many fields [1,2]. However, due to the broadcast nature, information is vulnerable to be intercepted by the eavesdroppers which are in the coverage area of the transmission. Security is an important factor in modern wireless communication networks. For the secure transmission of IoT (Internet of Things) [3,4,5] and crowdsensing [6,7] networks, conventional schemes based on computational complexity are to encrypt the data at the data link layer or the application layer, such as reputation management schemes [8] and privacy-preserving participant selection scheme [9]. Unfortunately, these schemes neither can prevent transmitted information from being cracked nor are suitable for power-constraint sensor devices in crowdsensing networks. At present, physical layer security provides a new paradigm that can effectively protect information from being eavesdropped by exploiting stochastic characteristics of wireless channels [10,11].

Maurer firstly put forward the idea that legitimate parties can extract secret key by exploiting channel characteristics in [12]. Subsequently, Hershey et al. [13] proposed that legitimate parties could use the reciprocity of the wireless channel to generate the secret key in Time Division Duplexing (TDD) systems. The independence and randomness of the reciprocal channel guarantee the security of the key. At present, most of the key-extracting methods are based on time-varying channels [14,15]. However, the wireless channel in some application scenarios is static or quasi-static, and the key generated with such methods is not suitable for encryption, because the key entropy or the key generation rate is too low. To improve the security performance, diversity technology based on multi-antenna system and relay system has attracted more and more attention [16,17,18,19,20]. However, a device in a low-cost wireless network, e.g., the sensor node in WSN (Wireless Sensor Network), is usually equipped with only one antenna. Furthermore, the physical-layer key generation method assumes that the eavesdropping device is placed outside the half-a-wavelength range of the legitimate receiver. However, this assumption has not been rigorously evaluated in the open literature, and it might be invalid in some practical scenarios [21], which do not experience extensive multi-path scattering. It is shown in [22] that in reality a strong correlation may be encountered between the main channel and the wiretap channel, even when the eavesdropper is located significantly more than half-a-wavelength away from the legitimate receiver.

Recently, the use of digital modulation encryption as a new physical layer security technology has attracted lots of attention. Because its security does not depend on the channel characteristics, this kind of physical layer encryption technology has inherent advantages in the communication security aspect under the static or quasi-static channel environment. In [23], Zang et al. proposed an encrypting scheme based on MSK (Minimum Shift Keying) modulator which possesses eight different structures. In this scheme, the legitimate transceiver and receiver synchronously change the structure of the modulator according to a set of random numbers generated from the *m* sequence. The eavesdropper cannot demodulate the received signal correctly without knowing the structure of the modulator. However, considering the high standardization of modern communication protocols, when the eavesdropper knows the modulator structure and part of the original text, the *m* sequence used to control the modulator structure may be deciphered by the eavesdropper. In [24], Husain et al. proposed a physical layer encryption scheme based on the diversity of 16QAM (Quadrature Amplitude Modulation) constellation mapping which was regarded as secret key and unavailable for eavesdroppers. This scheme can achieve the perfect secret proposed by Shannon and provide a promising prospect in the military area. However, this scheme is not suitable for highly standardized civilian communications. Ma et al. [25] proposed a scheme to secure communication with the aid of symbol rotation and artificial noise. Wang proposed to secure OFDM (Orthogonal Frequency Division Multiplexing) by two-stage chaos mapping and symbol rotation [26]. However, in those two schemes, only the phase of the constellation is changed, and the eavesdropper can obtain transmitting data by cryptanalysis.

Because chaotic signals have impulse-like auto-correlation and low cross-correlation values, many DCSK (differential Chaos Shift Keying) modulation technique for secure multi-user communication systems have been studied and evaluated [27,28]. Cooperative relaying and friendly jamming schemes have been recognized as a promising approach to enhancing the security. A comprehensive survey of the recent works on cooperative relaying and jamming techniques for securing wireless transmissions is provided in [29]. Artificial noise (AN) transmission is another effective approach to enhancing security provided that the instantaneous CSI (Channel State Information) of each eavesdropper is not available. A secrecy beamforming scheme, which exploits AN-aided to secure multiple-input single-output non-orthogonal multiple access (MISO-NOMA) transmission, is proposed in [19]. The secrecy capacities under various channel fading with AN are analyzed in [20,30,31]. Furthermore, Atallah et al. [32] proposed different protocols to foil the eavesdropper. Most of the recently proposed AN schemes are based on a hypothesis that the number of transmit antennas is larger than that of the receive antennas [20]. This strategy might fail when both legitimate parties are equipped with single antennas in some scenarios.

In this paper, a physical layer encryption technique based on artificial Rayleigh fading is proposed for the security of digital transmission of low cost single antenna wireless nodes in a static or quasi-static channels. The main idea of the proposed scheme is as follows. For the transmitter, each digitally modulated symbol is multiplied by a complex random variable used to imitate channel fading in wireless communications. For legitimate recipients, the identical complex random variable can be generated synchronously so that the transmitted information can be recovered correctly. For eavesdroppers, if they could not acquire synchronously the complex random variable, no information can be obtained. In this paper, a synchronous chaotic sequence generator is used to generate an uniformly distribution random sequence, and then a complex random variable whose envelope is Rayleigh distribution and whose phase is an uniformly distributed is generated by the transformation of the uniformly distribution.

The main contributions of this paper are as follows. (1) A physical layer encryption method is proposed. The legitimate parties in a static or quasi-static channel generate complex random variables (key) with a synchronous chaotic sequence, which is used to simulate the static or quasi-static channel into a Rayleigh fading channel. (2) A two-stage interleaver is introduced to make a synchronous chaotic sequence divided into four groups of irrelevant sequences. (3) The scrambling of chaotic sequences by the introduced two-stage interleaver significantly increases the difficulty of the eavesdropper’s cracking.

The rest of this paper is organized as follows. The proposed physical layer encryption scheme, including the structure of the two-stage interleaver and the generation process of complex Gauss random variables, is elaborated in Section 2. We derive the theoretical BER (bit error ratio) for the proposed communication system, and verify it by MATLAB in Section 3. The simulation results show that eavesdroppers cannot intercept any effective information under the condition of unknown (or unsynchronized) chaotic sequence and/or the structure of the two-stage interleaver. Finally, a summary is made in Section 4.

## 2. Secure Communication Scheme

In this paper, we assume that both legitimate parties are equipped with single antenna, and their communication protocols are open and standardized. We consider the secure transmission of the communication between the two parties in a static/quasi-static channel. In wireless communication, the amplitude and phase of the signal sent by the sender are randomly faded or fluctuated due to the random characteristics of the channel. If the channel estimation is not accurate enough, the SNR (signal-to-noise ratio) of the receiver may deteriorate dramatically and the demodulator cannot work properly, which results in a high BER. The most serious consequence is the inability to communicate, that means the receiver fails to obtain any information. Inspired by this, we propose the following encryption communication scheme. The training sequence used for channel estimation is sent according to the communication protocol, and the legitimate users or eavesdroppers can estimate the CSI accurately. For binary bitstreams that represent confidential information, the legitimate parties need to be encrypted and decrypted to prevent interception by the eavesdroppers. We emphasize here that both encryption process and decryption process are implemented in the physical layer. A synchronous chaotic generator is employed to generate random complex variables (also called the secret key) $h_{k}$, whose magnitude follows Rayleigh distribution and the phase follows an uniformly distribution within (-π,π), respectively. The binary bitstream to be transmitted is mapped into symbols $\left\{s_{k}\right\}$ after a digital modulator (e.g., 16QAM). Then, $s_{k}$ is multiplied by a random complex variable $h_{k}$ (also called encryption), which imitates the random fading in wireless communication. The legitimate receiver generates a secret key that is completely synchronized and consistent with that of the transmitter. The received symbol is divided by the key to recover the encrypted symbol (also called decryption). Thus, the digital demodulator can work properly. On the contrary, the eavesdropper uses the channel estimation of the training sequence in the static channel to demodulate the received symbols that have experienced the artificial random fading channel. Obviously, its demodulator cannot work properly, which results in high BER. Therefore, the secret information is hard to intercept.

**Figure 1:**
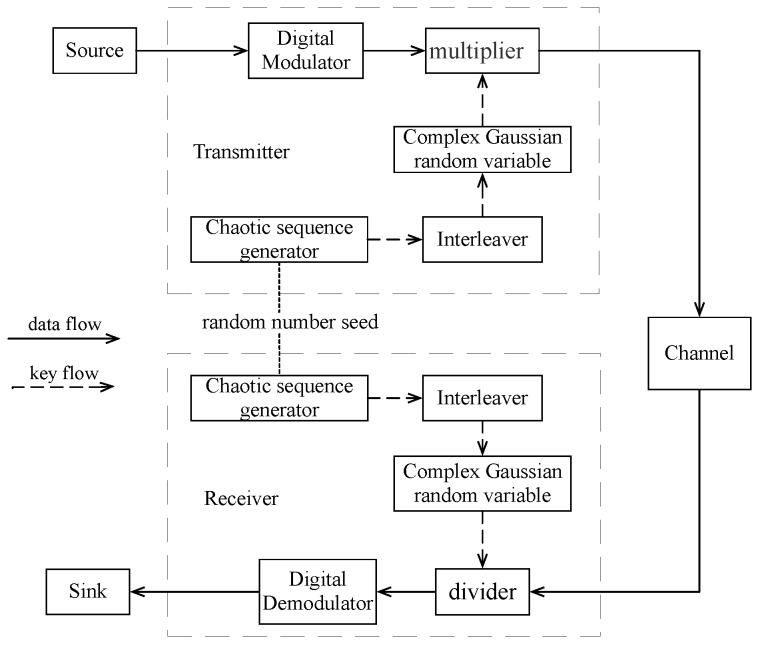
Block diagram of the proposed secure communication system with artificial Rayleigh fading and two-stage block interleaver (baseband only).

This section mainly introduces the baseband communication system with the secure transmission shown in Figure 1, including the chaotic sequence generator, the process of generating the complex random variable, and the structure and working mode of the two-stage block interleaver. The “key” in Figure 1 is composed of the mapping equation of chaotic sequence, the initial value and the number of iterations before chaotic generator enters into chaotic period. Furthermore, we assume that the chaotic generator of legitimate receiver is perfectly synchronized with that of the transmitter.

### 2.1. Generation of Random Complex Variables (Secret Key)

In wireless communications, Rayleigh [33] is the most common statistical model to describe the time-varying characteristics of the received envelope statistics of flat fading signals or independent multi-path components. In this paper, an artificial Rayleigh model is used to imitate channel fading. The fading coefficient, $h_{k}$, can be expressed as

(1) $$h_{k}=h_{k,R}+ıh_{k,I}=\mu_{k}e^{ı\theta_{k}}\;,$$

where $h_{k,R}$ and $h_{k,I}$ are two independent random variables of normal distribution with a mean value of 0 and a variance of $\frac{1}{2}$, respectively, such that the power of $h_{k}$ is equal to 1, i.e., $h_{k,R},h_{k,I}∼N\left(0,\frac{1}{2}\right)$ and $E[|h_{k}{|}^{2}]=1$. In Equation (1), $\mu_{k}$ and $\theta_{k}$ are the envelope and phase of $h_{k}$ respectively. Thus, $\mu_{k}$ obeys Rayleigh distribution, and $\theta_{k}$ obeys the uniformly distribution within (-π,π). The mean value of $h_{k}$ is 0, which means $E\left[h_{k}\right]=E\left[h_{k,R}+h_{k,I}\right]=0$. To increase the randomness of secret keys, the keys should be independent with each other, i.e.,

(2) $$\begin{array}{cc} E[h_{k}h_{l}^{*}] & =E\left[\left(h_{k,R}+ıh_{k,I}\right)\left(h_{l,R}-ıh_{l,I}\right)\right]=E\left[h_{k,R}h_{l,R}+h_{k,I}h_{l,I}\right]+ıE\left[h_{k,I}h_{l,R}-h_{k,R}h_{l,I}\right]=\delta_{kl}\;, \end{array}$$

where E[·] denotes expected operator, and ${\left(\cdot \right)}^{*}$ denotes conjugate operator.

To make it harder for eavesdropper to decipher, a complex random variable, $h_{k}$, is generated to change the envelope and phase of a digital modulated symbol $s_{k}$. Thus, the transmitted symbol, $x_{k}$, can be given by

(3) $$x_{k}=s_{k}h_{k}=s_{k}\mu_{k}e^{ı\theta_{k}},\;1\leq k\leq N\;,$$

where *N* represents the number of symbols to be transmitted. It is reasonable to assume that both $s_{k}$ and $h_{k}$ are independent with each other because they come from different sources. Moreover, due to the normalized fading power, $E[|h_{k}{|}^{2}]=1$, the power of the transmitted symbols, $E[|x_{k}{|}^{2}]$, can be expressed as

(4) $$E\left[|x_{k}{|}^{2}\right]=E\left[|s_{k}{|}^{2}\right]E\left[|h_{k}{|}^{2}\right]=E\left[|s_{k}{|}^{2}\right]\;.$$

This means that the encryption operation does not introduce additional power consumption.

We note that $h_{k}$ in Equation (1) is composed of two independent normal distribution random variables. It is well known that a standard normal distributed random variable can be transformed by two independent uniformly distribution random variables through the classic Box–Muller equation [34]. Let x,y∼U(0,1) and z∼N(0,1); the expression of Box–Muller equation is written as

(5) $$z=\sqrt{-2\ln x}\cos\left(2\pi y\right)\;.$$

Chaos is a deterministic pseudorandom process that occurs in nonlinear dynamic systems. This process is aperiodic, non-convergent and highly sensitive to initial values [35]. Owing to the sensitive dependence on initial conditions, it allows generating an infinite number of uncorrelated signals. In this paper, a Tent mapping [36] is chose to generate the uniformly distributed chaotic sequence $\left\{b_{i}\right\}$. The Tent mapping equation is written as

(6) $$b_{i+1}=2\beta \left(1-|b_{i}|\right)-1,\;\;i=1,2,\cdots$$

An appropriate parameter β in Equation (6) can guarantee that $\left\{b_{i}\right\}$ is a uniformly distributed in the range of (-1,1), and be set to 0.999 throughout this paper. A sampled chaotic sequence with length L=1000, and the initial seed $b_{1}\in \left(-1,1\right)$ is generated randomly. As we known, an ECDF (Empirical Cumulative Distribution Function) is the distribution function associated with the empirical measure of a sample in statistics. Therefore, an ECDF is introduced to measure the distribution of random sequences. Let $\left(x_{1},\cdots ,x_{n}\right)$ be IID (Independent, Identically Distributed) real random variables with the common cumulative distribution function F(t), then the ECDF is defined as

(7) $${\hat{F}}_{n}\left(t\right)=\frac{\mathrm{number}\;\mathrm{of}\;\mathrm{elements}\;\mathrm{in}\;\mathrm{the}\;\mathrm{sample}\leq t}{n}=\frac{1}{n}\sum_{i=1}^{n}1_{x_{i}\leq t}\;,$$

where $1_{A}$ is the indicator of event *A*.

**Figure 2:**
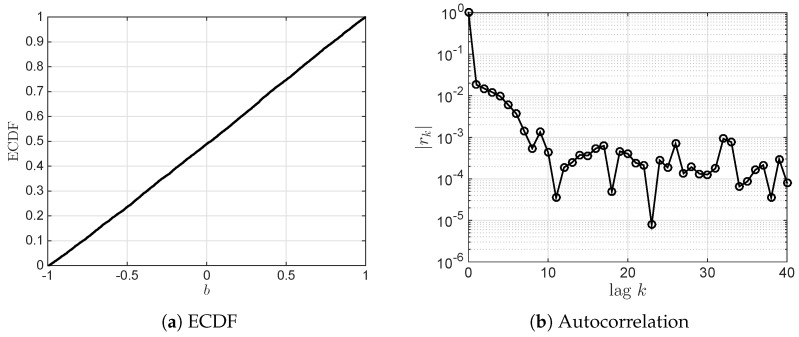
Empirical cumulative distribution function and absolute autocorrelation coefficient of a sampled chaotic sequence $\left\{b_{i}\right\}$ using Tent mapping equation, where functions cdfplot() and xcorr() in MATLAB are used to calculate ECDF and $r_{k}$, respectively.

As shown in Figure 2, ECDF of a sampled chaotic sequence is a straight line with slope 0.5. It indicates that $\left\{b_{i}\right\}\in \left[-1,1\right]$ obeys uniformly distribution. Furthermore, to verify its correlation, the autocorrelation coefficient is introduced and defined as [37]

(8) $$r_{k}=\frac{c_{k}}{c_{0}}\;,c_{k}=\frac{1}{L-k}\sum_{i=1}^{L-k}\left(b_{i}-\bar{b}\right)\left(b_{i+k}-\bar{b}\right)\;,\bar{b}=\frac{1}{L}\sum_{i=1}^{L}b_{i}\;.$$

Absolute autocorrelation coefficients of the first 41 lags of a sampled chaotic sequence, $|r_{k}|\;\left(k=\;0,1,\cdots ,40\right)$, are calculated and shown in Figure 2. It is noted that when the lag is greater than 1, $|r_{k}|$ is close to 0.01, and it stays around 0.001 when the lags are greater than 10. Therefore, it is reasonable to assume that uniformly distribution sequence $\left\{b_{i}\right\}$ is IID.

A new chaotic sequence $a_{i}∼U\left(0,1\right)$, which satisfies the condition of Box–Muller transformation, is obtained through a simple linear transformation defined as

(9) $$a_{i}=\frac{b_{i}+1}{2}\;.$$

According to Equations (1) and (5), we note that four independent and uniformly distributed random variables are required to generate a complex normal distribution random variable through Box–Muller transformation. Intuitively, four chaotic generators should be employed in a legitimate user. This greatly increases the complexity of both parties. To simplify the structure of the system, a two-stage block interleaver is introduced in our proposed secure scheme. With the aid of the interleaver, only one chaotic sequence generator is needed to produce four uniformly distribution random variables that satisfies the requirement of Equation (1), i.e., $h_{k,R}$ and $h_{k,I}$ are two independent random variables.

### 2.2. Two-Stage Block Interleaver

**Figure 3:**
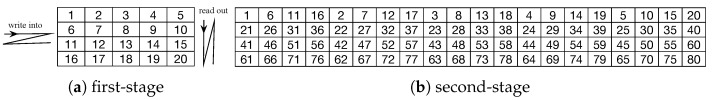
Two-stage block interleaver.

As mentioned in Section 2.1, the chaotic generator shown in Equation (6) could generate an uniformly distributed chaotic sequence $\left\{b_{i}\right\}$. Two subsets extracted from the chaotic sequence $\left\{b_{i},i=\;1,2,\cdots ,\right\}$, e.g., $\left\{b_{1},b_{9},b_{17},b_{25},b_{33},\cdots \right\}$ and $\left\{b_{5},b_{13},b_{21},b_{29},b_{37}\cdots \right\}$, can be considered as independent with each other if their cross-correlation is small enough. To generate four independent chaotic sequences from a chaotic generator, a two-stage block interleaver is proposed, and its structure is shown as Figure 3.

The numbers in Figure 3 represent the original order of chaotic sequence generated by the chaotic generator. The work mode of those two block interleavers is written in column-wise and read in line-wise. The output of the first-stage block interleaver is the input of the second-stage block interleaver. Thus, four independent chaotic sequences are obtained via the scrambling by the two-stage block interleaver, respectively, i.e.,

(10a) $$\begin{array}{cc} x_{1} & =\{b_{1},b_{21},b_{41},b_{61},b_{6},b_{26},b_{46},b_{66},\cdots ,b_{2},b_{22},b_{42},b_{62}\}\;, \end{array}$$

(10b) $$\begin{array}{cc} x_{2} & =\{b_{7},b_{27},b_{47},b_{67},b_{12},b_{32},b_{52},b_{72},\cdots ,b_{8},b_{28},b_{48},b_{68}\}\;, \end{array}$$

(10c) $$\begin{array}{cc} y_{1} & =\{b_{13},b_{33},b_{53},b_{73},b_{18},b_{38},b_{58},b_{78},\cdots ,b_{14},b_{34},b_{54},b_{74}\}\;, \end{array}$$

(10d) $$\begin{array}{cc} y_{2} & =\{b_{19},b_{39},b_{59},b_{79},b_{5},b_{25},b_{45},b_{65},\cdots ,b_{20},b_{40},b_{60},b_{80}\}\;. \end{array}$$

For each chaotic sequence in Equation (10), the adjacent offset is greater than 20. Hence, as can be seen in Figure 2, the absolute autocorrelation coefficient is less than 0.001. Similarly, for each pair of chaotic sequences $\left(x_{i},y_{i}\right),i\in \left\{1,2\right\}$, the offset is 12, thus its corresponding absolute cross-correlation coefficient is also less than 0.001. Therefore, four chaotic sequences via the proposed two-stage block interleaver meet the requirements of independence to generate a complex random key.

To improve the efficiency of generating random keys, two two-stage block interleavers, as shown in Figure 3, are introduced to perform read/write operations on those two interleavers in turn. When 80 chaotic numbers are written to the first Interleaver A, the subsequent 80 chaotic numbers are written to the second Interleaver B. When Interleaver A reads empty, Interleaver B must have been filled, and Interleavers A and B exchange read/write operation in turn. For each digital modulated symbol, four chaotic numbers are generated by the chaotic generator. Four independent chaotic numbers are outputted by the proposed two-stage interleaver, and then transformed by Equation (5), a complex random variable shown in Equation (1) is generated. Furthermore, another benefit of the introduced interleaver is that even if the eavesdropper synchronizes with the transmitter’s chaotic generator by some means, but if the structure of the interleaver is unknown, the eavesdropper still fails to generate a random key which is consistent with that of the transmitter. The consequence is that the eavesdropper cannot correctly demodulate the received symbols. Therefore, the introduced interleaver increases the difficulty of key cracking and further improves the security of communication.

### 2.3. Quantization of Complex Random Variables

**Figure 4:**
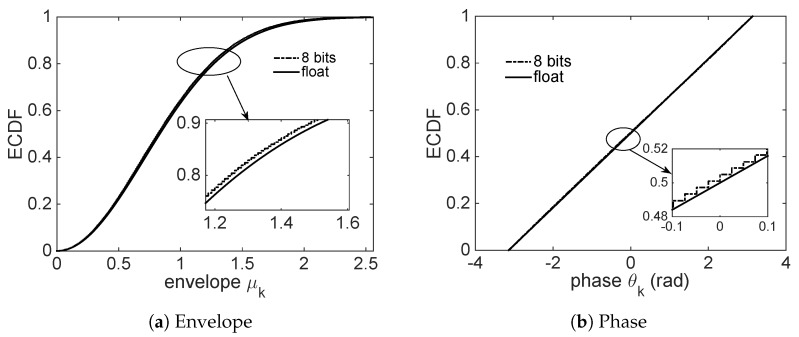
Empirical cumulative distribution function diagram of normalize complex Gauss random variable, in which solid line represents floating point operations, and dashed line represents 8-bit quantization operations.

The initial seed $b_{1}\in \left(-1,1\right)$ is randomly generated, the parameter β is set to 0.999 and a chaotic sequence of length 800,000 is generated, then its corresponding complex Gauss random variable sequence of length 200,000, $\left\{h_{k}\right\}$, is also generated via the proposed interleaver and the Box–Muller transformation. It is easy to calculate its magnitude $\left\{\mu_{k}\right\}$ and phase $\left\{\theta_{k}\right\}$, and their corresponding ECDFs are shown in Figure 4.

It can be seen in Figure 4 that the magnitude obeys the Rayleigh distribution and the phase obeys the uniformly distribution on (-π,π), which are completely consistent with the theory. In practical engineering, there is a quantitative problem because of the limitation of the finite word length of the digital-to-analog conversion device. Note that the aforementioned envelope of complex Gauss random variable obeys the Rayleigh distribution, and the power of $h_{k}$ is normalized to 1. Thus, the PDF (Probability Density Function) of the envelope, $f_{\mu }\left(x\right)$, is given by

(11) $$f_{\mu }\left(x\right)=2xe^{-x^{2}}\;,$$

and its cumulative distribution function (CDF) is written as

(12) $$\mathrm{Pr}\left(\mu \leq x\right)=\int_{0}^{x}f_{\mu }\left(t\right)dt=1-e^{-x^{2}}\;.$$

According to Equation (12), it is easy to know that the probability of envelope greater than 2.55 is 0.14%. Thus, a n=8 quantized bits are used to represent size of envelope, whose range is [0,2.55], i.e., the envelope amplitude of the corresponding 1 bits is 0.01. In Addition, for envelope amplitude greater than 2.55, it is truncated to 2.55. Similarly, an 8-bit quantization is also used in the phase on (-π,π), and the corresponding phase of one bit is $\frac{\pi }{128}$ radians. Whether the quantified complex Gaussian random variable as a key satisfies the one-time pad depends on the analysis of the key characteristics. Here, whether the quantified complex Gauss random variable obeys strictly IID is analyzed. For complex Gauss variables, satisfying the correlation shown in Equation (2) means they are independent with each other. We use the simulation to verify the correlation of quantified random variable. In this simulation, 200,000 samples of complex gaussian random variables $\left\{h_{k}\right\}$ are generated, whose envelope and phase are quantified by 8-bit, as shown in Figure 4. It can be seen that the 8-bit quantization is close enough to the floating point operations.

Furthermore, to verify the independent expression in Equation (2), a normalized correlation coefficient is introduced and defined as:

(13) $$r_{q,k}=\frac{\sum_{i=1}^{L-k}\left(h_{q,i}-\bar{h_{q}}\;\right){\left(h_{q,i+k}-\bar{h_{q}}\;\right)}^{*}}{\sum_{i=1}^{L}\left(h_{q,i}-\bar{h_{q}}\;\right){\left(h_{q,i}-\bar{h_{q}}\;\right)}^{*}}\;,\bar{h_{q}}=\frac{1}{L}\sum_{i=1}^{L}h_{q,i}\;,$$

where $\left\{h_{q,i}\right\}$ is a quantized complex Gaussian random variable with length of *L*, the superscript ${\left(\cdot \right)}^{*}$ denotes complex conjugate operator, and *k* is the lag.

**Figure 5:**
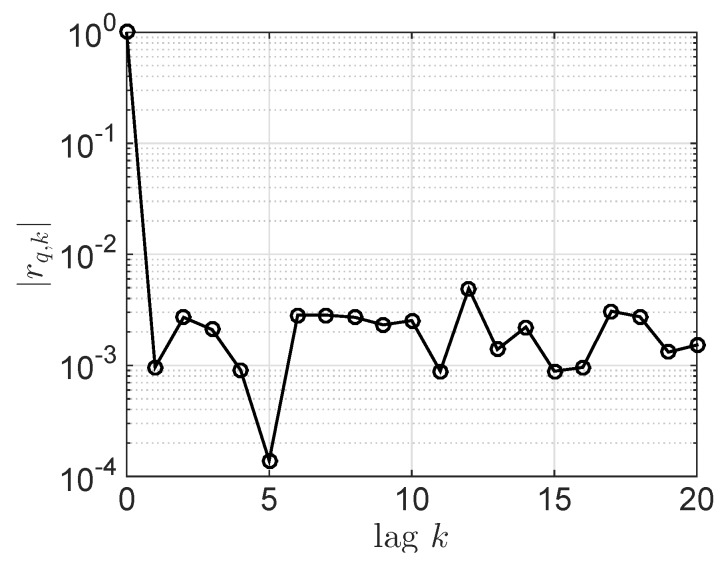
Absolute value of normalized correlation coefficient of complex Gauss random variables with 8-bit quantization.

In this simulation, the absolute value of normalized correlation coefficient with lags *k*, $|r_{q,k}|$, are calculated and shown in Figure 5. It can be seen that $|r_{q,k}|$ is less than 0.01 when the offset is greater than 1, which means that the quantified complex random variables are IID. Furthermore, it is noted that the chaotic sequence has a strong sensitivity to the initial seed, and the imperceptible changes of the initial seed will make the chaotic sequence to change greatly. Therefore, legitimate parties can simultaneously change the initial seed to increase difficulty of deciphering the chaotic sequence by eavesdroppers. The analysis of key space and decoding time based on the two-stage chaotic map OFDM security transmission are presented in [38]. Similar to this literature, our proposed secure transmission scheme also has a large key space and long crack time.

## 3. Performance Analysis and Simulation

### 3.1. Theoretical BER

Assuming that the power spectral density of white noise n(t) is $\frac{N_{0}}{2}$, the bandwidth of signal is *B* and the interval of symbol is $T_{s}$. $E_{b}$ denotes the bit energy and $E_{s}$ represents the symbol energy. If shape filter satisfies $T_{s}=\frac{1}{B}$, and Gray code mapping is used in digital modulation, then the relationship between bit signal to noise ratio $\gamma_{b}$ and symbol signal to noise ration $\gamma_{s}$, BER (Bit Error Ratio) $P_{b}$ and SER (Symbol Error Ratio) $P_{s}$ are give by

(14) $$\begin{array}{c} \left\{\begin{array}{c} \gamma_{b}=\frac{E_{b}}{N_{o}}=\frac{E_{s}}{N_{o}\log_{2}M}=\frac{\gamma_{s}}{\log_{2}M}\;,\\ P_{b}≅\frac{P_{s}}{\log_{2}M}\;. \end{array}\right. \end{array}$$

Table 6.1 in [33] lists the common digital modulations and their corresponding SER/BER when coherent demodulation is used, e.g., SER of the correlated demodulation under AWGN for rectangular *M*QAM(M>4) is

(15) $$P_{s}\left(\gamma_{s}\right)=1-{\left(1-\frac{2(\sqrt{M}-1)}{\sqrt{M}}Q\left(\sqrt{\frac{3\gamma_{s}}{M-1}}\;\right)\right)}^{2}$$

and BER is

(16) $$P_{b}\left(\gamma_{b}\right)≃\frac{4}{\log_{2}M}Q\left(\sqrt{\frac{3\gamma_{b}\log_{2}M}{M-1}}\;\right)\;,$$

where Q(x) function is defined as

(17) $$Q\left(x\right)=\int_{x}^{\infty }\frac{1}{\sqrt{2\pi }}e^{-\frac{t^{2}}{2}}dt=\frac{1}{2}\mathrm{erfc}\left(\frac{x}{\sqrt{2}}\right)\;,$$

and erfc(x) is the complementary error function.

Next, we derive the theoretical BER expression of the legitimate receiver under Rayleigh fading channel. As mentioned above, the envelope of complex Gauss random variable, $\mu_{k}$, obeys Rayleigh distribution and its power $E[\mu_{k}^{2}]=1$. Thus, $\lambda_{k}=\mu_{k}^{2}$ obeys the exponential distribution with a parameter of 1, i.e., its PDF is given by

(18) $$f_{\lambda }\left(\lambda_{k}\right)=e^{-\lambda_{k}}\;.$$

In the static additive white Gaussian noise (AWGN) channel, the symbol received by the legitimate user is

(19) $$r_{k}=s_{k}h_{k}+n_{k}\;,$$

where complex Gaussian white noise $n_{k}∼\mathrm{CN}\left(0,N_{0}\right)$. After the symbol is encrypted, its instantaneous SER (or BER) is $\lambda_{k}\gamma_{s}$ ($\lambda_{k}\gamma_{b}$), and the average SER (or BER) is obtained by statistical averaging, i.e.,

(20a) $$\begin{array}{cc} \bar{P_{s}}\left(\gamma_{s}\right) & =\int_{0}^{\infty }P_{s}\left(\lambda \gamma_{s}\right)e^{-\lambda }d\lambda \end{array}$$

(20b) $$\begin{array}{cc} \bar{P_{b}}\left(\gamma_{b}\right) & =\int_{0}^{\infty }P_{b}\left(\lambda \gamma_{b}\right)e^{-\lambda }d\lambda \end{array}$$

Combining Equation (20) and Table 6.1 in [33], the BER expressions of BPSK, 4QAM and 16QAM with proposed encryption scheme are given by

(21) $$\begin{array}{c} \bar{P_{b}}\left(\gamma_{b}\right)=\left\{\begin{array}{c} \frac{1}{2}\left(1-\sqrt{\frac{\gamma_{b}}{1+\gamma_{b}}}\;\right)\;\;\;\;\;\;\;\;\;\;\;\mathrm{BPSK}/\mathrm{QPSK}/4\mathrm{QAM}\\ \frac{1}{\log_{2}M}\left(1-\sqrt{\frac{\gamma_{b}\log_{2}M}{\csc^{2}\left(\frac{\pi }{M}\right)+\gamma_{b}\log_{2}M}}\;\right)\;\;\;\;M\mathrm{PSK}\left(M>4\right)\\ \frac{2}{\log_{2}M}\left(1-\sqrt{\frac{3\gamma_{b}\log_{2}M}{2\left(M-1\right)+3\gamma_{b}\log_{2}M}}\;\right)\;\;\mathrm{Rectangular}\;\;M\mathrm{QAM}\left(M>4\right) \end{array}\right. \end{array}$$

### 3.2. Simulations and Analysis

**Figure 6:**
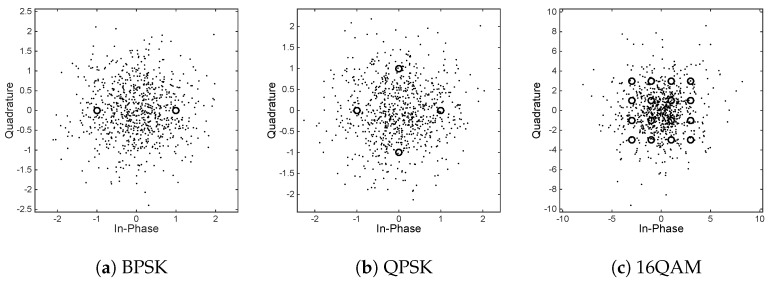
Constellations of the received 800 symbols with bit signal to noise ratio $\gamma_{b}=20$ dB, where its corresponding digital modulation constellation is represented by the marker “o”.

In this section, we carry out Matlab (ver. R2014b) simulation on the system shown in Figure 1 to verify the BER performance of legal user and eavesdropper, respectively. The channel is assumed to be static AWGN channel and is invariant in the whole simulation. The parameter in the Tent mapping β is set to 0.999, and initial seed of chaotic sequence $b_{1}$ is a random number in (-1,1). Figure 6 shows the encrypted constellation of BPSK, QPSK, and 16QAM, respectively, when $\gamma_{b}=20$ dB. It can be seen that the random variation of its phase and amplitude leads to the random distribution of the digital modulated symbol. AMC (Automatic Modulation Classification) [39] and DMC (Digital Modulation Classification) [40] technologies for modulation recognition based on time periodicity of constellation change are bound to fail because their constellation does not show periodic changes in time. Therefore, the proposed transmission scheme has good security.

The chaotic generator at the legitimate receiver is assumed to be synchronized perfectly, i.e., the generated complex random variables are identical with that of the transmitter. Based on Equation (19), the legitimate user decrypts the received symbol $r_{k}$ before demodulation, i.e., the decrypted symbol $\hat{s_{k}}$ is given by

(22) $$\hat{s_{k}}=\frac{r_{k}}{h_{k}}=s_{k}+\frac{n_{k}}{h_{k}}\;.$$

**Figure 7:**
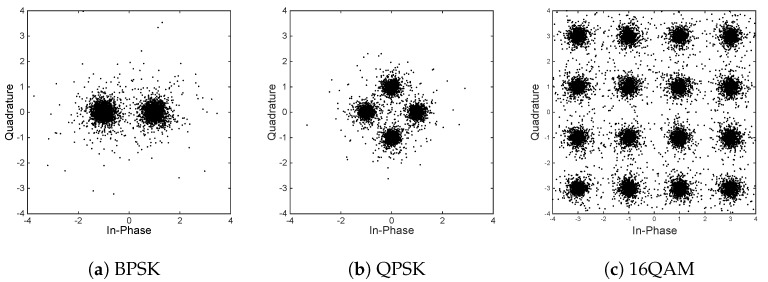
Constellation (10,000 symbols) after demodulation of the legitimate user with $\gamma_{b}=20$ dB.

Figure 7 shows the decrypted constellation of the received symbols (10,000 symbols) with $\gamma_{b}=\;20$ dB. Because the envelope of the complex normal random variable obeys Rayleigh distribution, there are lots of very small envelope amplitude values, i.e., there are many symbols near the 0+0ı neighborhood, as shown in Figure 6. The term $n_{k}/h_{k}$ in Equation (22) produces a relatively large value, i.e., the decryption operation amplifies significantly the complex Gauss noise $n_{k}$. Figure 7 shows the constellation of 10,000 decrypted symbols (the range of quadrature and in-phase components is limited to (−4,4)), and there are many symbols of magnitude far exceeding that of the corresponding digitally modulated constellation. In Figure 7, it can be seen that the influence of noise amplification is obviously messy in the vicinity of the digital modulation constellation. However, it is still possible to distinguish the type of its modulation, and most of the symbols are able to be demodulated correctly. BER will be further decreased if the constellation after decryption is clearer. Otherwise, it will be more indistinct. Meanwhile, if the eavesdropper does not get the secret key, its constellation is very indistinct and the demodulator cannot work properly.

**Figure 8:**
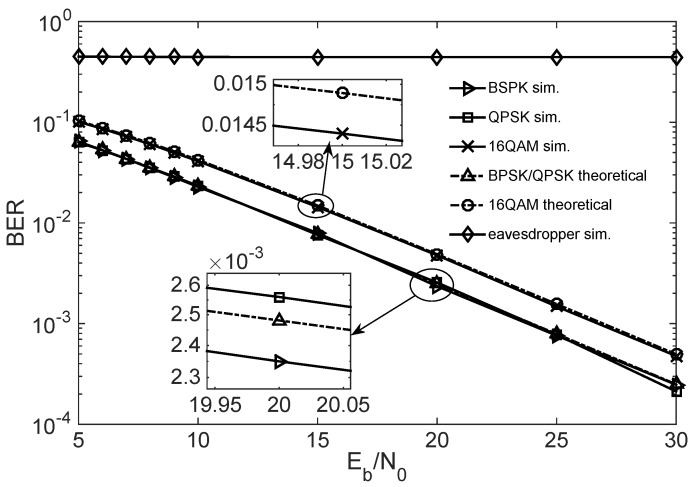
BER of the legal user and eavesdropper with various digital modulations.

Figure 8 shows the BER performances of the legitimate receiver and eavesdropper, respectively. As shown in Figure 8, BER of the legitimate receiver decreases with the increase of bit SNR $E_{b}/N_{0}$, and the simulation results fully conform to the theoretical derivation colorred in Equation (21). Meanwhile, because the eavesdropper does not recover the key consistent with the sender, its BER will not decrease with the increase of the bit SNR, which is about 0.5. As a result, the eavesdropper does not intercept any effective information.

### 3.3. Secrecy Capacity

Considering the case of a complex, flat-fading channel with the receiver having perfect knowledge of the channel state, the ergodic capacity of such a channel is given by [41]:

(23) $$C=E\left[\log_{2}\left(1+\frac{{\left|h\right|}^{2}P}{\sigma_{w}^{2}}\right)\right]\;,$$

where E(·) denotes the expectation operation, *P* is a fixed transmit power, $\sigma_{w}^{2}$ is the noise variance, $P/\sigma_{w}^{2}$ is called SNR, and the expectation is taken over the gain *h* of the channel. In this paper, the AWGN channel is stationary and ergodic, and bit SNR is denoted as $\gamma_{b}$. As described in Section 3.1, the power of a complex Gaussian random variable obeys the exponential distribution, and its PDF, f(λ), is given by Equation (18). Therefore, the capacity is calculated as follows.

(24) $$C=\int_{0}^{+\infty }\log_{2}\left(1+\lambda \gamma_{b}\right)f\left(\lambda \right)d\lambda =\int_{0}^{+\infty }\log_{2}\left(1+\lambda \gamma_{b}\right)e^{-\lambda }d\lambda =-\frac{1}{\ln2}\mathrm{Ei}\left(-\frac{1}{\gamma_{b}}\right)e^{\frac{1}{\gamma_{b}}}\;.$$

In Equation (24), the exponential integral function, Ei(z), is defined as $\mathrm{Ei}\left(z\right)=\int_{-\infty }^{z}e^{t}/tdt$, where the principal value of the integral is taken [42].

At the same time, we can see from the simulations in Section 3.2 that the eavesdropper’s error rate is close to 0.5, as shown in Figure 8. This implies that the eavesdropper’s channel capacity is close to 0. The secrecy capacity is the channel capacity between Alice and Bob (legitimate users), minus the channel capacity between Alice and Eve (eavesdropper). Therefore, the secrecy capacity is obtained and given by

(25) $$C_{s}=C_{AB}-C_{AE}=-\frac{1}{\ln2}\mathrm{Ei}\left(-\frac{1}{\gamma_{b}}\right)e^{\frac{1}{\gamma_{b}}}\;.$$

## 4. Conclusions

In this paper, we have proposed a scheme for physical layer security transmission of single antenna node in wireless crowdsensing networks under static channel. In our scheme, the chaotic sequence generator with Tent mapping is used to generate random complex variables. The modulated symbol is multiplied by the complex random variable (encryption) to imitate the Rayleigh fading of the channel at the transmitting end. The received symbol is divided by the identical complex random variable (decryption) to recover the transmitted message before the digital demodulation at the receiving end. The eavesdropper is unable to intercept any effective information in the case of the unsynchronized the chaotic sequence and/or the unknown structure of the two-stage block interleaver, while the legitimate user can still transmit in security under a certain BER. With the introduction of artificial fading, the phase random rotation of the complex random variable does not cause the increase of BER. However, the variation of the modulated symbol power caused by the envelope fluctuation of the complex random variable , makes the symbol SNR fluctuate. When the envelope is less than 1, the power of the modulation symbol sent to the channel is reduced, that is, the signal-to-noise ratio is reduced, leading to the increase of the bit error ratio. This means reducing reliability to improve safety. Rayleigh fading is one of the worst fading channels. If we want to reduce BER while keeping security, we can also consider *m*-Nakagami fading. It is well known that Nakagami fading degenerates to Rayleigh fading when m=1, and degenerates to no fading channel when m=∞. Therefore, it is possible to choose an appropriate *m* value to take both reliability and security into account.
